# Detecting Selection on Protein Stability through Statistical Mechanical Models of Folding and Evolution

**DOI:** 10.3390/biom4010291

**Published:** 2014-03-07

**Authors:** Ugo Bastolla

**Affiliations:** Centro de Biologia Molecular Severo Ochoa, CSIC-UAM, Madrid E-28049, Spain; E-Mail: ubastolla@cbm.csic.es; Tel.: +34-911-964-633

**Keywords:** protein folding, misfolding, negative design, protein evolution, natural selection

## Abstract

The properties of biomolecules depend both on physics and on the evolutionary process that formed them. These two points of view produce a powerful synergism. Physics sets the stage and the constraints that molecular evolution has to obey, and evolutionary theory helps in rationalizing the physical properties of biomolecules, including protein folding thermodynamics. To complete the parallelism, protein thermodynamics is founded on the statistical mechanics in the space of protein structures, and molecular evolution can be viewed as statistical mechanics in the space of protein sequences. In this review, we will integrate both points of view, applying them to detecting selection on the stability of the folded state of proteins. We will start discussing positive design, which strengthens the stability of the folded against the unfolded state of proteins. Positive design justifies why statistical potentials for protein folding can be obtained from the frequencies of structural motifs. Stability against unfolding is easier to achieve for longer proteins. On the contrary, negative design, which consists in destabilizing frequently formed misfolded conformations, is more difficult to achieve for longer proteins. The folding rate can be enhanced by strengthening short-range native interactions, but this requirement contrasts with negative design, and evolution has to trade-off between them. Finally, selection can accelerate functional movements by favoring low frequency normal modes of the dynamics of the native state that strongly correlate with the functional conformation change.

## 1. Introduction

Proteins bridge physics and biology. On the one hand, they are amazing molecular machines that obey the laws of statistical mechanics. On the other hand, they are evolving machines that are shaped by selective and mutational forces acting on the populations in which they evolve. Simple models of protein folding thermodynamics allow the identification of these evolutionary forces and to better understand and model molecular evolution. In turn, modeling evolution allows a better understanding of the properties and constraints acting on protein thermodynamics. This article aims to review the mutual relationship between models of protein folding and models of protein evolution, in particular how we can use evolutionary reasoning for detecting selective forces that target protein folding thermodynamics and the intrinsic dynamics of protein native states. It does not pretend to be exhaustive, but rather, it will focus on a simple, contact-based model of protein folding and on the results that I and collaborators have gathered with the help of this model.

## 2. Contact-Based Model of Protein Folding

Proteins are complex molecules, formed by thousands of atoms kept together by quantum mechanical interactions. The solvent in which they reside, water molecules and ions, plays a key role in determining their statistical mechanical properties. This complexity needs to be reduced to a simple representation, if we want to make quantitative predictions. The representation adopted in this work is based on contact matrices [1]: for each pair of residues at positions *i* and *j* along the polypeptidic chain, *C_ij_* equals one if the residues are in contact and zero otherwise. We define two residues to be in contact if a pair of heavy atoms belonging to the two residues are closer than 4.5 Å. Since contacts with |*i* − *j*| ≤ 2 are formed in almost all structures, they do not contribute to the free energy difference between the native and the misfolded ensemble, and we set *C_ij_* = 0 if |*i* − *j*| ≤ 2. This representation has some important weak points: it is not continuous; it does not include any repulsion term; and it does not allow angular dependencies, such as those that occur in hydrogen bonds and aromatic interactions. However, its discreteness simplifies the computations and facilitates obtaining analytic insight.

A contact matrix, *C_ij_*, represents a mesoscopic state in which the degrees of freedom, such as the solvent, exact side chain positions, *etc.* are averaged out. We model its free energy as the sum of contact interactions, *E*(*C*, *A*) = ∑*_i_*_<_*_j_ C_ij_U*(*A_i_*, *A_j_*), which depends on the nature of the amino acids in contact, *A_i_* and *A_j_*, and on 210 contact interaction parameters, *U*(*a*, *b*), that express the average interaction between amino acids *a* and *b* at a given temperature, such as those determined as a statistical potential by Miazawa and Jernigan [2]. A limitation of this energy function is that it does not allow for representing atomic clashes, hydrogen bonds and secondary structures; therefore, we cannot apply it to generic conformations, but only to conformations that fulfill these strong interactions, such as protein structures deposited in the protein databank. Despite these limitations, contact matrices are useful in that they provide the simplest model of protein folding, commonly adopted in lattice models and applicable to experimentally determined protein structures [3,4,5].

We have to keep in mind that the conformational entropy associated with a contact matrix, *S*(*C_ij_*), decreases with the number of contacts, since mesoscopic structures with more contacts have more constraints. This conformational entropy is difficult to evaluate analytically, and it is almost always neglected in the calculations. Karplus and coworkers showed through normal modes calculations and quantum mechanical calculations that the conformational entropy of a folded protein is large (of the order of 35 cal/(mol × K)) per residue, but its contribution to protein denaturation can be neglected, since the conformational entropy of the denatured state can be modeled as the sum of the entropy, due to the existence of different conformations (of the order of 4 to 6 cal/(mol × K) per residue) plus the weighted sum of the vibrational entropy in each conformation; the latter term almost exactly balances the entropy in the native state, although differences in these terms may have relevant consequences for ligand binding and for the thermodynamic effect of mutations [6]. Computational approaches inspired by this idea, reviewed by Doig and Sternberg [7], estimate the loss of conformational entropy upon folding from the reduction in the number of accessible rotamers of side chains and yield similar values compatible with experimental results. Nevertheless, differences in entropy between folded states with different compactness may be important for yielding a correct statistical mechanical picture [8,9,10,11].

A protein may exist in several mascroscopic states separated by free energy barriers, the most studied ones being: (1) the native state where it performs its biological function; in this state, the protein is usually folded into a well-defined three-dimensional structure, except for the important case of natively unfolded proteins [12], which will not be considered here; (2) the unfolded state, dominated by conformational entropy; (3) misfolded states, where they are folded into a non-native structure that is not functional. For small proteins that fold with two-states thermodynamics [13], only the natively folded and the unfolded state are relevant. However, the thermodynamics of medium and large proteins (typically, larger than 90–100 residues) present more than two states. These compact states may be folding intermediates that often resemble the molten globule state with native secondary structure and loosely packed side chains [14,15], but they may also lay outside the folding pathway from the unfolded to the native state; in this case, they can act as a kinetic trap and reduce the rate at which functional proteins are formed [16,17]. They may even trigger pathological protein aggregation, as in amyloidosis [18], but the formation of structured aggregates, such as amyloid fibers, is more difficult to model and so will not be considered here. It has been suggested that selective pressure to reduce protein misfolding is a major evolutionary force that targets the frequency of incorrect translations produced by theribosome [19].

For the sake of simplicity, we neglect the conformational entropy of the folded native state, estimating its free energy as $G_{\text{nat}}(C^{\text{nat}},A)\approx \sum_{i<j}C_{ij}^{\text{nat}}U(A_{i},A_{j})$, and we neglect contact interactions in the unfolded state, estimating its free energy as *G_U_* ≈ −*TLS_U_*, where *T* is the temperature in units in which *k_B_* = 1, *L* is chain length and *S_U_* is the conformational entropy per residue of an unfolded chain. It has been proposed that the misfolded state, consisting in the ensemble of compact, but wrongly folded conformations, is described by the Random Energy Model (REM) [20], which approximates the energy with a Gaussian random variable [21,22,23]. In a similar spirit, we can go beyond the REM by computing the free energy of the misfolded ensemble from the partition function of all possible compact contact matrices, obtaining the analytic approximation [24]:

(1) $$\begin{array}{ll} G_{\text{misfolded}} & \equiv -T\log\left(\underset{C}{\text{∑}}e^{-\sum_{i<j}C_{ij}U(A_{i},A_{j})/T+S(C)}\right)\\ & \approx \langle E\rangle -\frac{\langle {\left(E-\langle E\rangle \right)}^{2}\rangle }{2T}+\frac{\langle {\left(E-\langle E\rangle \right)}^{3}\rangle }{6T^{2}}-LS_{C}T \end{array}$$

where *LS_C_* is the logarithm of the number of compact contact matrices, 〈.〉 represents the average over the set of alternative compact contact matrices of *L* residues (To derive Equation (1), we write the sum over all contact matrices grouping those with the same number of contacts *N_C_* = ∑*_i_*_<_*_j_ C_ij_*, and we distinguish a homopolymer energy (all contact interactions equal to 〈*E*〉 /*N_C_*) and a heteropolymer contribution, writing exp(−*G*/*T*) = ∑*_N_C__* exp (− 〈*E*〉 /*T* + *S*(*N_C_*)) ∑*_C:N_C__* exp(−*βz*), with *z* = (∑*_ij_ C_ij_U_ij_* − 〈*E*〉/*N_C_* and *β* = *N_C_*/*T*. We then approximate $\log(\sum_{C}\exp(-\beta z)\approx S_{C}+\log\langle \exp(-\beta z)\rangle \approx \frac{1}{2}\langle {\left(\beta z\right)}^{2}\rangle -\frac{1}{6}\langle {\left(\beta z\right)}^{3}\rangle )$. Terms of higher order in *z* can be neglected, since their contribution is of order 1/*β*. We also assume for simplicity that the conformational entropy, *S*(*C_ij_*), is approximately the same for all compact structures, and it can be neglected for computing free energy differences.

With respect to the usual REM approximation, the above formula also contains the third moment of the energy This fact changes the nature of the freezing transition of the misfolded ensemble. As for the REM, the above model has a critical temperature, *T_c_*, at which the conformational entropy vanishes: $S=-(\partial G/\partial T)=S_{C}-\frac{\langle {\left(E-\langle E\rangle \right)}^{2}\rangle }{2T_{c}^{2}}+\frac{\langle {\left(E-\langle E\rangle \right)}^{3}\rangle }{3T_{c}^{3}}=0$. Below *T_c_*, the ensemble freezes into a finite number of thermodynamically relevant contact matrices, and the free energy maintains the same value as at *T* = *T_c_*. If the third centered moment of the energy, 〈(*E* − 〈*E*〉^3^〉, is negative (the system is more attractive than if it were Gaussian;, the freezing temperature is higher than for the REM, *i.e.*, freezing is facilitated. However, if the third moment is positive and the total conformational entropy, *S_C_*, is large, the conformational entropy is always positive, and the freezing transition does not take place. Instead, the specific heat vanishes at the critical temperature $T_{c}=\frac{\langle {\left(E-\langle E\rangle \right)}^{3}\rangle }{\langle {\left(E-\langle E\rangle \right)}^{2}\rangle }$ [24], and the misfolded ensemble has a second order phase transition reminiscent of a coil-globule *E* ransition. This model also shows that the freezing temperature is larger when the average contact energy is more negative, *i.e.*, proteins are more hydrophobic, and when the chain length is large. Therefore, we expect that the selection for negative design becomes stronger for more hydrophobic and for longer proteins. These expectations are verified by the statistical analysis reported below.

Putting together these free energy estimates, we obtain the free energy difference between the native and the non-native states above the freezing temperature, Δ*G*(*A*, *C*^nat^) = *G*_nat_ − *G*_misfolded_ − *G*_unfolded_, as:

(2) $$\begin{array}{cc} \Delta G(A,C^{\text{nat}}) & =TL(S_{C}+S_{U})+\underset{i<j}{\text{∑}}\left(C_{ij}^{\text{nat}}-\langle C_{ij}\rangle )U(A_{i},A_{j}\right)\\ & +\underset{i<j,k<l}{\text{∑}}(\langle C_{ij}\rangle -{\langle C_{ij}\rangle }^{2})\frac{U(A_{i},A_{j})U(A_{k},A_{l})}{2T}\\ & -\underset{i<j,k<l,m<n}{\text{∑}}F_{\text{ijklmm}}\frac{U(A_{i},A_{j})U(A_{k},A_{l})U(A_{m},A_{n})}{6T^{2}} \end{array}$$

where *F_ijklmm_* = 〈(*C_ij_* − 〈*C_ij_*〉 (*C_kl_* − 〈*C_kl_*〉 (*C_mn_* − 〈*C_mn_*〉)〉 [24]. The free energy depends on the mean contact frequency and on the correlation and skewness of the contacts.

In this work, we use the contact interactions parameters that were determined in [25,26] by requiring that the contact energy function assigns optimal stability to all proteins represented in the protein databank. In several cases, we have tested that the results are robust when we use different contact interaction parameters, such as those determined by Godzik, Kolinsky and Skolnick [27]. Averages with respect to the ensemble of compact protein conformations are computed by threading the protein sequence against all possible fragments of protein structures present in the protein databank (PDB), a procedure that is known in the bioinformatic jargon as “gapless threading” [28,29].

## 3. Modeling Selection on Protein Folding Thermodynamics

Traditionally, molecular evolution studies have examined the rate of substitution of amino acids in protein sequences without considering their structural context. This situation has become, since less than two decades ago [30,31,32,33,34,35,36,37,38,39,40,41,42,43,44], due to the availability of models of protein folding, simple enough to allow detailed simulations that aim to “bring molecules back into molecular evolution” [45], recently reviewed in [46].

Following Goldstein and other researchers (see, for instance, [47] for a recent presentation), we model the fitness associated to a protein as the time spent by the protein in the correctly folded state that is the target of natural selection,

(3) $$f(A,C^{\text{nat}})=\frac{{\text{e}}^{-\Delta G(A,C^{\text{nat}})/T}}{1+{\text{e}}^{-\Delta G(A,C^{\text{nat}})/T}}$$

In the above model, we assume that selection only acts on the stability of the native state and disregard other aspects important for function, such as protein intrinsic dynamics. There are two reasons for this assumption: first, it is much simpler to predict protein stability than protein dynamics or, in general, protein “function”; second, protein dynamics is correlated with the topology of the native structure, as the elastic network models shows [48], so that the targeting protein structure also influences protein dynamics.

Evolution takes place in a population. Whereas a mutation is a “microscopic” event arising in a single individual, its fixation in the population (substitution) is a macroscopic event. Two main factors influence the evolutionary process and, consequently, the stability that an evolving biomolecule can achieve: the effective population size and the mutational process, in particular mutation rates and mutation bias. As reviewed below, we found that population size and mutation bias have an important effect on protein folding thermodynamics. Recombination can be very important as a source of evolutionary innovation, as well, but we do not consider it here, because it is more complex to analyze.

An important property of the above fitness function is that it presents a neutral regime at low temperature. In this case, the fitness takes approximately the value one when Δ*G* < 0 and zero when Δ*G* > 0, *i.e.*, it tends to a binary value in which a protein is either non-viable or equally fit as other viable proteins. In this neutral regime, the stability achieved by the protein is almost independent of population size as in the neutral model by Kimura. In Kimura's model, the rate at which neutral substitutions are fixed in a population of *N* individuals evolving with neutral mutation rate *μ* is independent of population size, since *Nμ* neutral mutations arise in a time unit, and the probability that one of them is fixed is 1/*N* [49]. Taverna and Goldstein [39] showed that, in the nearly-neutral regime of the fitness function Equation (3), the stability attained by a protein coincides with the minimum stability for which the protein is viable (*f* ≈ 1), *i.e.*, Δ*G* ≈ 0, since Δ*G* ≈ 0 is the most probable stability of viable proteins in sequence space. They proposed that this property of the neutral regime explains why natural proteins are only marginally stable, despite it not being difficult to engineer mutations that increase their stability

At high enough temperature, however, the fitness function Equation (3) presents a non-neutral regime in which differences in stability have important consequences. This regime can be analytically studied in the limit of a very small mutation rate in which different mutations do not interfere. In this limit, Moran's model of the evolutionary process allows one to compute the probability that a mutation is fixed in the population and becomes a substitution as a function of the ratio between the fitness of the wild-type and the mutant [50]:

(4) $$P_{\text{fix}}(\text{wt}\to \text{mut})=\frac{1-f_{\text{wt}}/f_{\text{mut}}}{1-{\left(f_{\text{wt}}/f_{\text{mut}}\right)}^{N}}$$

It has been noted by Sella and Hirsch that in this limit of a small mutation rate, the evolutionary process is equivalent to a Monte Carlo stochastic process in sequence space and that this process converges to a stationary Boltzmann-like distribution in which the probability to observe a sequence, *A*, is proportional to the exponential of its logarithmic fitness *φ* = log(*f*), *P*(*A*) ∝ exp(*N_φ_*(*A*)), *i.e.*, there is a perfect analogy between molecular evolution and statistical mechanics with the effective population size, *N*, playing the role of the inverse of temperature [51]. In this non-neutral regime, larger populations achieve higher fitness and, therefore, better stability of their macromolecules. For Equation (3), *φ*(*A*) = − log(1 + exp(Δ*G*(*A*)/*k_B_T*)), and the distribution of stability in the space of the protein sequences that are visited by evolution is:

(5) $$P(\Delta G)\propto \exp(-N\log(1+\exp(\Delta G))\approx \{\begin{array}{cc} \exp(-Ne^{\Delta G}) & \Delta G\ll -1\\ \exp(-N\Delta G) & \Delta G\gg 1 \end{array}$$

where we used units, such that *k_B_T* = 1. Note that the approximation *P*(Δ*G*) ∝ exp(−*N*Δ*G*/*kT*) is only valid in the first steps of evolution when the protein is unstable, whereas when Δ*G*/*kT* becomes very negative, either because the temperature is low or because evolution optimized protein stability, the fitness saturates to its maximum possible value *f* = 1 and becomes almost independent of Δ*G*, and we enter a neutral regime in which protein stability is almost independent of population size. The above equation has to be corrected to take into account the mutation process, in particular the bias to mutate to hydrophobic or polar amino acids, which influences the fitness attained in evolution and the corresponding protein stability, see below.

**Figure 1:**
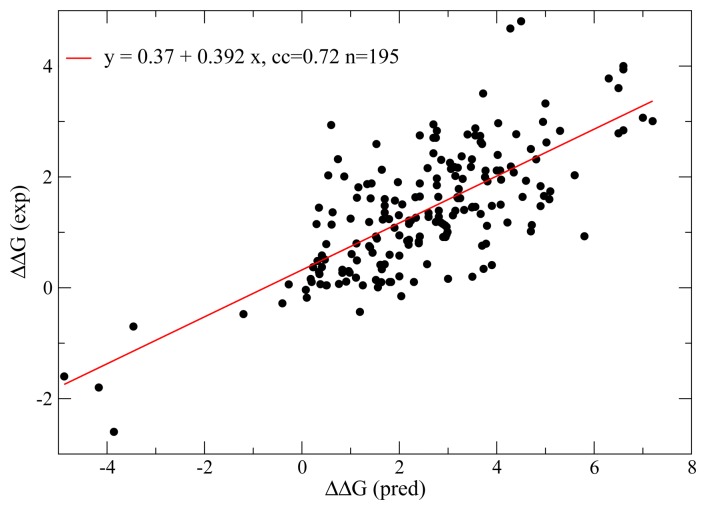
Computational predictions of the thermodynamic effect of mutations with the contact energy parameters of [26]. The 195 mutants were taken from [52].

By computationally predicting Δ*G* from the protein sequence, *A*, and the target native structure, *C*^nat^, we can map the genotype into the phenotype of the model protein and perform quantitative studies and simulations of molecular evolution. This mapping is not accurate enough to reliably predict the effect of individual mutations, but there is a good correlation between the effect of mutations predicted through this model and those experimentally measured (the correlation coefficient is 0.72 for a set of 195 mutants that fold with two-state thermodynamics [52], see Figure 1), so that the statistical signals that arise from these simulations are credible.

## 4. Validations and Limitations of the Model and Assessment of Neutrality

The modeling scheme described above, in which the fitness is assumed to be dependent on protein folding stability, has been adopted by several groups with different points of view. The computational study of heteropolymer folding through lattice models led to the proposal of simple criteria for fast folding and native state stability, based either on the energy gap between the native conformation and misfolded conformations [53] or on the gap between the folding temperature and the glass temperature [54]. These criteria inspired the first computational models of the evolution of stable and fast-folding model proteins [30,31,32], which allowed for the studying of the robustness of model proteins against mutations and to reproduce the fact that natural proteins tolerate a large number of sequence changes, yet maintain a similar structure [33,34,35,36,37,38,39]. These models have then been applied to the contact matrices of real protein structures, and it has been shown that they reproduce the qualitative properties of the natural evolution of their sequences [40,41,42,43], so that they get closer to the important goal of building models of molecular evolution that are aware of structural constraints and, yet, simple enough for phylogenetic inferences [44,45,46].

Despite the success of these models, it is well known in the field that a fitness function only based on folding stability is a poor approximation of the real selection process, which acts on protein “function” (whatever it means). In particular, the ability of proteins to establish specific molecular interactions and their native dynamics may be important targets for selection (see the last section of this review), even more than the stability of the folded state, which has little relevance for the important class of natively unfolded proteins [12]. Another important limitation of these models is that they tend to be too tolerant of mutations with respect to natural protein evolution: using a model similar to the ones discussed here, Goldstein observed that the selective coefficients simulated by these models (*i.e.*, the difference of the logarithm of the fitness between the wild-type and the mutant that gets the fixation) tend to be very small and that the evolutionary rate *dN*/*dS* (*i.e.*, the rate between non-synonymous substitutions, which change the amino acid, and synonymous ones, which maintain it, expressing the acceptance rate of mutations in the protein sequence) are high and almost independent of population size, as expected in neutral evolution [55].

An important remark with respect to this observation is that the neutrality of these models depends on temperature, as noted above: the fitness function Equation (3) tends to a binary value at low temperature, *i.e.*, *f* ≈ 1 if Δ*G* < 0 and *f* ≈ 0 if Δ*G* > 0, yielding a neutral fitness landscape in which the evolution is almost independent of population size and the selective coefficients for viable proteins (Δ*G* < 0) are very small: Δ log *f* ≈ exp(Δ*G*/*kT*)ΔΔ*G*/*kT* ≈ 0, where ΔΔ*G* is the thermodynamic effect of mutations. Nevertheless, if the temperature is high, these models are much less neutral, and the properties of evolved proteins depend on population size, as was found, for instance, in the simulations reported below.

## 5. Detecting Selection through Null Models Based on Physics and Population Genetics

The strategy that we follow here for detecting selection on some protein property relies on comparing two physically-based models, one that includes the effect of selection on the target property and another one that only considers selection on a lower order property (null model). For instance, when we investigate selection on stability with respect to misfolded states (the so-called negative design), we compare the predictions based on misfolding stability with the predictions of a null model that only considers the stability of the native state with respect to the unfolded state. The model based on misfolding stability predicts that the average interaction energies of contacts that are frequent in the misfolded ensembles should be higher, whereas the null model predicts that the average energies of native contacts should be low. To compare these predictions, we divide pairs of residues into four classes: (A) native and frequent, (B) native and infrequent, (C) non-native and frequent and (D) non-native and infrequent. According to the null model, no difference is expected between class A and B and between class C and D, whereas observed interaction energies show significant differences.

This procedure for detecting stability based on the predictions of physical models can be complemented with methods based on biological models that focus on substitution rates and intrapopulation variability. Nucleotide sequences can be used to infer rates for synonymous (those that only change the messenger RNA, but not the coded amino acid) and non-synonymous substitutions separately [56], although this estimate is reliable only if the compared species are not too diverged (otherwise, synonymous substitutions saturate) nor too close (the rate of non-synonymous substitutions has been observed to be higher at short time separation [57], an observation that has been attributed to the fixation of ancestral polymorphisms upon speciation [58]). Since selection on the amino acid sequence is expected to be stronger than selection on the RNA sequence, it is expected that the synonymous substitution rate, *dS*, is higher than the non-synonymous rate, *dN*. Genes showing values of *dN*/*dS* larger than typical values, but smaller than one, are usually interpreted as a hint that the gene is under relaxed selection, either because the effective size of the population in which it evolves is small or because the phenotypic consequences of its incorrect functioning are not severe. Values of *dN*/*dS* > 1, *i.e.*, accelerated evolution at the amino acid level, are usually interpreted as an indication that the gene is under positive selection, for instance due to a change in its function or a change in the environment. Since the *dN*/*dS* > 1 criterion is a very strong requirement, more sensitive tests have been developed, such as the McDonald and Kreitman test [59], which compares *dN*/*dS* to the analogous value for intraspecies polymorphisms. However, this test has been criticized for not being able to separate adaptation events from compensatory substitutions, which should be frequent in evolution: the model described by Equation (4) reaches an equilibrium state in which detrimental substitutions that decrease the fitness and compensatory substitutions that increase it almost exactly balance [51].

## 6. Positive Design: Protein Folding Potentials

In the previous sections, I briefly presented the models of protein folding stability (statistical mechanics in structure space, for a given sequence) and protein evolution (statistical mechanics in sequence space, for a given target structure) on which this work is based. I will now show that these models allow one to quantify how natural selection acted on several aspects of protein folding, starting from positive design, *i.e.*, the evolutionary forces that strengthen the interactions formed in the native state.

As first realized several years ago by Finkelstein and coworkers [60], the distribution of amino acids of a protein family that has maximum entropy conditioned to a given value of stability is a Boltzmann-like distribution, such as the one presented in Equation (5). This argument helps to understand why elements of protein architecture have a Boltzmann-like statistics. This analogy between the frequency of structural elements and the Boltzmann distribution, justified on an empirical basis rather than on evolutionary grounds, had been previously exploited to derive statistical potentials for protein folding, such as the contact interaction parameters, *U*(*a*, *b*), by measuring how the frequency of pairs of amino acids of type *a* and *b* that are in contact in structures from the PDB deviates from a null model that assumes the absence of interactions: contact parameters are estimated as *U*_stat_(*a*, *b*) = log (*P*(*C_ij_* | *A_i_* = *a*, *A_j_* = *b*)/*P*_null_(*C_ij_*)) [2,61]. Different parameters are obtained adopting different null models, called reference states in the literature on this subject. The above formula can be derived approximating the amino acid distribution as a product of pairwise terms, *P*(*A*_1_ ⋯ *A_L_*) ≈ ∏*_ij_ P_ij_* (*A_i_*, *A_j_*), and determining the distribution of maximal entropy with the constraint of fixed average native energy *E̅* = ∑*_A_ P*(*A*_1_ ⋯ *A_L_*) ∑*_ij_ C_ij_U*(*A_i_*, *A_j_*), *i.e.*, considering only positive design (Minning, Porto and Bastolla, Pairwise amino-acid distributions from structurally constrained protein evolution, preprint). The contact interaction parameters, *U*_stat_(*a*, *b*), extracted with the above Boltzmann-like formula present a very strong correlation with the *U*(*a*, *b*) parameters used in this review [25], which were optimized in such a way that experimentally known native structures have maximum stability with respect to alternative structures obtained, threading their sequence against all other structures in the PDB. The strong correlation between parameters obtained in these two very different ways (*r* = 0.97, Minning, Bastolla and Porto, Pairwise amino-acid distributions from structurally constrained protein evolution, preprint) support the view that statistical potentials derived from database analysis or optimization criteria reflect relevant physical interactions underlying protein evolution.

A further interesting support to this view comes from the recent work by Lui and Tiana [62], who applied the method developed by Morcos *et al.* [63] to obtain residue coupling that can be related to the residue interaction energy. They showed that, when model protein sequences are generated by computationally optimizing sequences that fold in a target conformation with an empirical contact interaction energy, the contact interaction matrix can be reliably back-calculated with the method of [63]. Furthermore, they computed pairwise interaction parameters from multiple alignments of four families of natural proteins and showed that these parameters allow one to predict the thermodynamic effect of the mutations of the studied proteins with a correlation coefficient ranging from 0.65 to 0.89, providing further evidence of the link between protein stability and evolution. Of course, these correlations would not be expected under a mutational model of evolution that does not take into account positive design.

## 7. Site-Specific Amino Acid Distributions

A similar reasoning allows one to analytically compute site-specific distributions (profiles) of amino acids at different positions in a protein family. If we approximate *P*(*A_1_* ⋯ *A_L_*) as the product of single site distributions, *P*(*A*_1_ ⋯ *A_L_*) *≈* Π*_i_ P_i_* (*A_i_*), we can compute *P_i_*(*A_i_*) as the distributions that maximize the entropy in sequence space for a given value of stability, Δ*G*. To simplify this computation, I and coworkers adopted the hydrophobic approximation, which consists in approximating the contact interaction parameters with their main spectral component, which is related to hydrophobicity [64]:

(6) U(a,b)≈ϵh(a)h(b)

where *ϵ* < 0 and *h*(*a*) is correlated with several empirical hydrophobicity scales [65]. In this way, the energy transforms into the quadratic form *E* = *ϵ*∑*_ij_ C_ij_h_i_h_j_*, with *h_i_* = *h*(*A_i_*), and we can analytically determine the sequence that minimizes the energy for a fixed value of $\sum_{i}h_{i}^{2}$ and fixed average hydrophobicity, constraints imposed in order to limit the free energy of the misfolded ensemble. This is the sequence whose hydrophobicity profile, *h_i_* (a sequence signature), is proportional to the effective connectivity (EC), *c_i_* [66], a structural signature, in turn strongly correlated with the principal eigenvector of the contact matrix [65]. The condition that the stability of the protein is fixed can be then substituted by the simpler condition that the average hydrophobicity is proportional to the EC, $\bar{h_{i}}=\sum_{a}h(a)P_{i}(a)=\alpha c_{i}+b$. The distribution, *P_i_*(*a*), can then be computed as the distribution of maximal entropy subject to the constraint on its mean value. The result is a Boltzmann-like distribution, *P_i_*(*a*) ∝ exp(−*β_i_h* (*a*)). In the absence of selection, *P_i_*(*a*) would be the distribution given by the mutational process. Therefore, *P_i_*(*a*) is the distribution with minimum Kullback–Leibler divergence from the mutational distribution, *P*_mut_(*a*), *i.e.*, *P_i_*(*a*) ∝ *P*_mut_(*a*) exp(−*β_i_h*(*a*)), where the selection coefficient, *β_i_*, expresses the strength of natural selection at each position (the largest is |*β_i_*|, the more the distribution deviates from the one induced by mutation), and it can be determined by imposing the constraint ∑*_a_ h*(*a*)*P_i_*(*a*) = *αc_i_* + *b*. We have verified that this distribution is in very good agreement with the site-specific distributions obtained through simulated evolution with stability constraints [36,41], and it is in good agreement with the distribution that is obtained aligning sites of proteins with a known structure that have similar values of effective connectivity [66]. For a given protein family, the maximum likelihood fit of the observed profile, *f_i_*(*a*), to the above equation allows for the determining of the 21 parameters, *P*_mut_(*a*) (one of these parameters is given by the normalization condition), *α* and *b*, and to compute the exponent, *β_i_*. Note that *β_i_* depends on the mutational distribution, *P*_mut_(*a*). For instance, if mutations favor hydrophobic amino acids, selection will be stronger at exposed positions where selection favors hydrophilic residues. Conversely, if mutations favor hydrophilic amino acids, selection will be stronger at bulk positions, where hydrophobic residues are preferred [67].

### 7.1. Relationship between Chain Length and Positive Design

**Figure 2:**
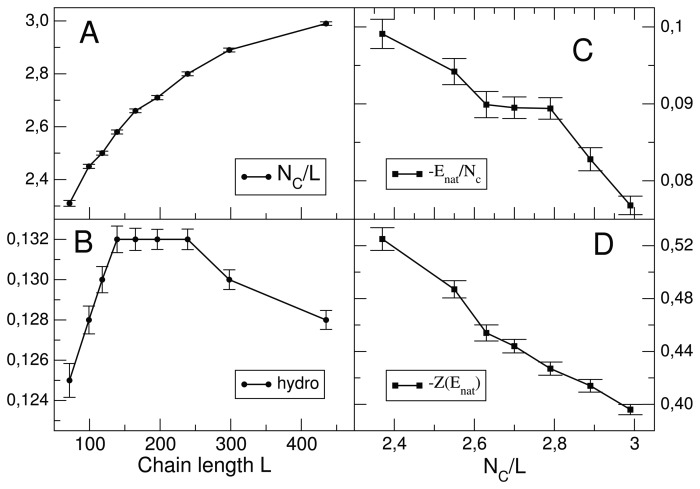
The number of contacts per residues, *N_C_*/*L*, increases with chain length (**A**), but the mean hydrophobicity reaches a maximum and then decreases for very long proteins (**B**). The predicted native energy per contact (**C**) and Z score of the native energy (D) increase with the number of contacts per residues, *NC*/*L*, *i.e*., native contacts become weaker and less optimized for more compact and longer proteins. Same data, as in [68], are used, consisting of 4,528 non-redundant proteins with a known structure.

Surface residues form fewer contacts than bulk residues. For globular proteins, the surface-to-volume ratio decreases with chain length as *L*^−1/3^. Therefore, longer proteins tend to have more contacts per residue: *N_c_*/*L* ≈ *c* (1 − *bL*^−1/3^) (see Figure 2A), and they can more easily compensate for the loss of conformational entropy upon folding, which is proportional to *LS_U_*. This observation led us to predict that proteins with a larger number of contacts per residue, and in particular, longer proteins, need to optimize their native contacts less in order to achieve the same level of stability If only the unfolded ensemble is thermodynamically relevant, as for proteins that fold with two-states thermodynamics, it holds Δ*G*/*L* ≈ ∑*_i_*_<_*_j_* $C_{ij}^{\text{nat}}U(A_{i},A_{j})-TS_{U}=\langle U(A_{i},A_{j})|C_{ij}^{\text{nat}}\rangle N_{c}^{\text{nat}}/L-S_{U}$, where $\langle U(A_{i},A_{j})|C_{ij}^{\text{nat}}\rangle =\sum_{ij}C_{ij}^{\text{nat}}U(A_{i},A_{j})/\sum_{ij}C_{ij}^{\text{nat}}$ is the mean energy of native contacts.

As we have seen above, when the physical temperature is low or Δ*G* is very negative, so that the fitness, *f*, is close to saturation, there is a neutral evolutionary regime in which we expect that proteins achieve only the marginal stability that allows their functioning. In this regime, we expect that the absolute value of $\langle U(A_{i},A_{j})|C_{ij}^{\text{nat}}\rangle$ decreases with *N_c_*/*L* or, which is the same, with chain length; in other words, individual native contacts are expected to be weaker for longer proteins. This prediction has been verified for a representative set of of proteins in the PDB [68]; see Figure 2C. Not only the average value, but also minus the Z score of native interactions with respect to all possible pairwise interactions, decreases with *N_c_*/*L*, *i.e.*, native interactions are less optimized; see Figure 2D. Conversely, as we saw above, for longer proteins negative design becomes more demanding, since the freezing temperature of the misfolded ensemble increases with protein length. Consistently, we find that the average hydrophobicity, 〈*h*〉, first increases with chain length, since the number of bulk *versus* surface residues increases, but then it reaches a maximum and decreases, *i.e.*, very long proteins tend to be less hydrophobic [68], which has the effect of reducing the stability of the misfolded ensemble (see Figure 2B).

## 8. Negative Design

Negative design, named in this way by Berezovsky and coworkers [69] and Horowitz and coworkers [70], is the selective force that tends to destabilize contacts that occur frequently in the ensemble of misfolded conformations. Negative design acts on sequence composition, disfavoring both very hydrophobic sequences in which the mean contact energy is strongly negative and very polar or charged sequences in which the mean contact energy is strongly positive, since they would not fold. Negative design also acts on sequence order, disfavoring those combinations of amino acids that stabilize frequent contacts.

To investigate selection on sequence composition, we divide amino acids into three classes: hydrophobic {L, I, V, F, Y, C, W, M}, polar and charged {D, E, G, S, N, K, R} and others {A, T, H, Q, P}, and we compared observed frequencies to a null model that takes into account that the sum of frequencies is one [24]. We found that selection favors two types of amino acid compositions: those in which a relatively large frequency of hydrophobic residues coexist with a large frequency of polar residues, and those in which both hydrophobic and polar residues are depleted. In the first kind of composition, the effective repulsion between polar and charged residues due to desolvation penalties and electrostatic repulsion compensates for the attraction between hydrophobic residues, perhaps by selectively destabilizing frequent contacts and correlated contacts, as described in the next section. This effect is stronger for longer proteins, in accordance with the expectation that selection for negative design is stronger in long proteins. In the second type of composition, both the frequencies of hydrophobic and polar amino acids are small, thus reducing the number of both potentially stabilizing and destabilizing interactions. Compositions in which the frequency of polar residues is large and the frequency of polar residues is small are strongly underrepresented, most probably due to positive design, since these compositions are typical of disordered proteins that are not present in the dataset that we examined (proteins that have been crystallized in the PDB). The opposite composition, in which a large hydrophobic frequency is not compensated by polar residues, is also underrepresented, probably due to negative design.

**Figure 3:**
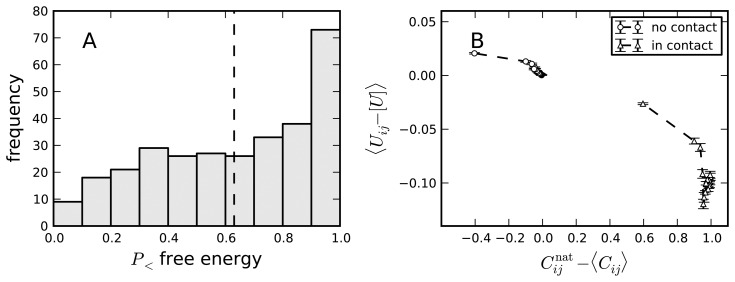
(**A**) For each protein in a large set, we compute the probability that the free energy of the misfolded ensemble is larger for the real sequence than for 100 randomly reshuffled sequences with the same composition. The results show that for most natural proteins, the misfolded ensemble is destabilized. (**B**) The average energy of a contact decreases with its nativeness index, $C_{ij}^{\text{nat}}-\langle C_{ij}\rangle$. In particular, short-range contacts with large 〈*C_ij_*〉 are less attractive. Reproduced with permission from [24] (Wiley^©^).

Since the entropy loss associated with a contact, *C_ij_*, is a decreasing function of the contact range *l* = |*i* − *j*|, short-range contacts occur more frequently, and they are weakened by a negative design. However, to observe this effect, we have to discount the effect of positive design by separating contacts that occur in the native state from those that do not. We define the contact frequency energy score (CFES) as the correlation between contact frequency, 〈*C_ij_*〉, and contact energy, *U*(*A_i_*, *A_j_*), distinguishing the set of native and non-native contacts. We observe that the CFESs are positive, *i.e.*, native contacts that are short range are associated with energies that are, in general, higher than for long-range native contacts [24]. The same conclusion is found if we compare non-native contacts that are short range with non-native contacts that are short range. If we reshuffle the sequence, maintaining its composition, we can see that this pattern is destroyed, *i.e.*, the values of the CFESs are strongly significant, which suggests that they are due to selection for negative design (see Figure 3). This pattern is mainly due to anticorrelations in the hydrophobicity of residues at neighboring positions along the protein chain.

Negative design also acts on contact correlations. We distinguished pairs of contacts, *C_ij_C_kl_*, involving only two (*kl* = *ij*), three (*ij*, *ik*) and four different indexes, and measured the correlation coefficient between contact correlations 〈*C_ij_C_kl_*〉 − 〈*C_ij_*〉 〈*C_kl_*〉 and energy products *U*(*A_i_, A_j_*)*U*(*A_k_, A_l_*), which we call the contact correlation energy score (CCES2, CCES3 and CCES4, respectively). Also in this case, we have to distinguish native and non-native contacts. All the CCESs are positive and significantly higher than their reshuffled version, which indicates that the second moment of the energy of misfolded conformations is higher than expected based on composition and native contacts alone, suggesting that natural selection is able to improve negative design by acting on contact correlations, as well [24].

The *Z*scores of the CFES and CCES with respect to shuffled sequences are significantly larger for native contacts, which are fewer and stronger, than for non-native contacts. These Z scores are larger for proteins with more negative interaction energy (*i.e.*, hydrophobic composition), as well as for longer proteins, in agreement with the expectation that these proteins experience stronger selection for negative design [24].

## 9. Selection on Protein Folding Rates

**Figure 4:**
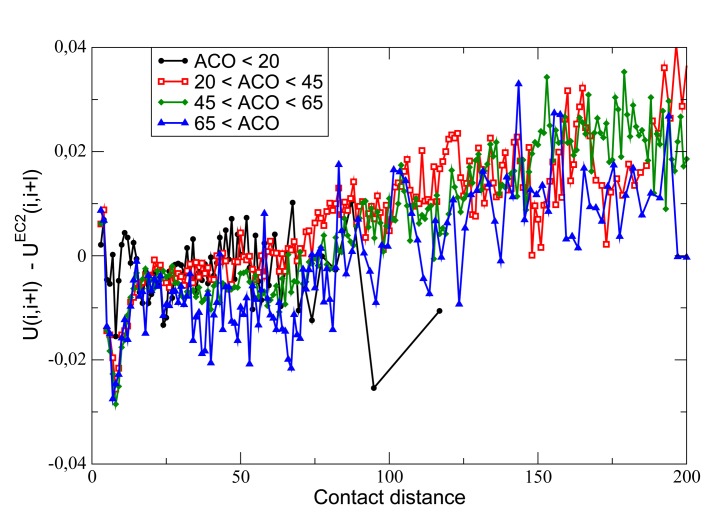
Excess energy of contacts, *i.e.*, the difference between the interaction energy of the natural sequence and the one of the sequence with optimal stability, *versus* contact distance |*i* − *j*|. One can see that short-range contacts are more attractive than expected on a stability ground, and this effect increases with chain length and with the absolute contact order (ACO). Reproduced with permission from [74] (Wiley ^©^).

Plotkin and coworkers developed a model that predicts that the protein folding rate increases when contacts that are short range along the sequence (*i.e.*, |*i* − *j*| is small), and, therefore, are more easily formed, interact more strongly, *i.e.*, *U*(*A_i_*, *A_j_*) is more negative [71,72,73]. This model predicts a positive correlation between the sequence difference of native contacts |*i* − *j*| and their interaction energy, *U*(*A_i_*, *A_j_*). However, we have seen in the previous section that negative design promotes a negative correlation between |*i* − *j*| and *U*(*A_i_*, *A_j_*), since increasing the energy of frequent contacts with small |*i* − *j*| destabilizes misfolded conformations. These two design principles cannot be achieved at the same time, which induces frustration in protein evolution. Nevertheless, despite the signal for negative design being stronger, the signal for selection on the protein folding rate can be detected from a statistical analysis of the PDB [74]. This analysis shows that short-range native contacts tend to have high energy, and the energy decreases with contact range, consistent with negative design, but it attains a minimum at |*i* − *j*| ≈ 8. For longer ranges, the energy increases with |*i* − *j*|. This trend cannot be attributed either to positive or to negative design. If we adopt a method similar to the one described above and described in more detail in [24] to design the hydrophobicity profile that guarantees optimal stability to the target native structure, taking into account both positive and negative design, we can estimate whether the interaction energy of a contact is higher in the real sequence than in the optimal sequence. This excess interaction energy tends to be significantly negative for short-range contacts, as shown in Figure 4. In other words, short-range contacts tend to be stronger than predicted based on folding stability, in agreement with Plotkin's model. Moreover, this effect is larger for proteins characterized by larger absolute contact order $\text{ACO}=\sum_{ij}C_{ij}^{\text{nat}}|i-j|/\sum_{ij}C_{ij}^{\text{nat}}$, a structural parameter that is negatively correlated with the protein folding rate [75]. Structures with large ACO tend to fold more slowly, and they are expected to be subject to stronger selective pressure for sequence features that accelerate the folding rate, such as the strong short-range contacts described in Plotkin's theory [74].

## 10. Influence of Mutation Bias and Population Size

**Figure 5:**
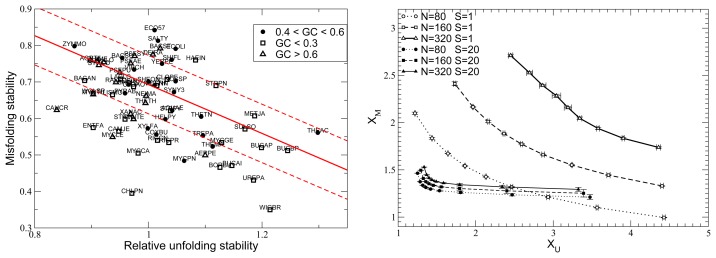
Relationship between stability against unfolding, *X_U_*, and stability against misfolding, *X_M_*. (**A**) An inverse relationship between the two kinds of stabilities is observed in orthologous bacterial proteins. (**B**) The same relationship in simulated protein evolution. Each line corresponds to a different population size, *N*, and neutrality parameter, *S*; each point represents a mutation bias measured as the GC content that would be attained with mutation alone. Mutations favoring GC produce less hydrophobic proteins, which are more stable against misfolding, but less stable against misfolding, moving leftwards in the horizontal direction. Stability increases with population size *N*, but it becomes less dependent on both population size and mutation bias for large neutrality parameter *S*.

Due to the structure of the genetic code, proteins coded by genes that are rich in the bases, adenine and thymine (in particular, T at the second codon position), tend to be hydrophobic, while proteins coded by genes rich in guanine and cytosine tend to be hydrophilic (in particular, genes coding for disordered proteins are GC-rich). This particularity establishes a deep relationship between the mutation process and protein folding thermodynamics. We tested this relationship with simulations of protein evolution subject to different mutation biases, finding that mutation bias towards AT produces proteins that are less stable against misfolding, but more stable against unfolding [67] (see Figure 5).

We tested this relationship through a statistical analysis of the predicted folding thermodynamics properties of orthologous proteins present in the genome of different bacteria [76]. Intracellular bacteria are characterized by mutation bias towards AT, as well as by reduced effective population sizes, in that they have to undergo severe population bottlenecks upon transmission to a new host. We found that their proteins are characterized by reduced stability against misfolding with respect to the orthologous proteins of their free living relatives. Consistently with this result, it was observed that intracellular bacteria express the chaperonin, DnaK [77], at very high level, which assists the folding of proteins that present misfolding problems; in particular, it binds to exposed hydrophobic patches and sequesters them, preventing protein aggregation. Overexpression of DnaK recovers a large fraction of the fitness that is lost upon experimental bottlenecks of bacterial populations transmitted from one plate to another [78], suggesting that a large part of this fitness loss is due to protein folding problems.

The case of intracellular bacteria is interesting in that two features of their evolution conspire to reduce the stability against the misfolding of their proteins: the small effective population size and the mutation bias towards AT (hence, towards hydrophobic proteins). To gain insight into the interplay between these two properties, we simulated protein evolution with a fitness function that separates stability with respect to the unfolded ensemble and with respect to the misfolded ensemble. We evaluate these two kinds of stability through the variables, *x_U_* (native energy minus conformational entropy) and *x_M_* (normalized energy gap between the native state and the misfolded ensemble), which we normalize with respect to the sequence in the PDB. This setting allows for highlighting the fact that these two kinds of stabilities respond differently to mutation bias and population size. For this study, we use the following fitness function:

(7) $$f(x_{U},x_{M,}S)=\{\begin{array}{ll} \frac{1}{1+x_{U}^{-S}+x_{M}^{-S}} & x_{U}>0\wedge 0x_{M}>0,\\ 0 & \text{otherwise}. \end{array}$$

The parameter, *S*, has the role of a neutrality parameter: if it is large, the fitness tends to a binary variable, and the value of fitness attained in evolution is independent of population size (see Figure 5B).

**Figure 6:**
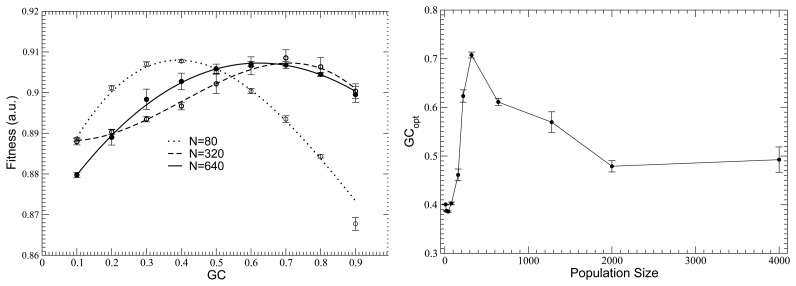
(**Left**) For fixed population size, fitness, and, hence, protein stability, attains a maximum as a function of mutation bias, measured as the GC content that would be attained under mutation alone. The three curves have been vertically shifted to make the intra-curve comparison clearer. (**Right**) This optimal mutation bias, GC_opt_, depends on the population size; it favors AT for a small population size, GC for intermediate population size and the absence of bias (GC = 0.5) for a very large population size. Reproduced from [79], copyright of the authors.

This model shows that the fitness attained in evolution depends not only on population size, as it was known, but also on mutation bias [79]. Namely, for a fixed population size, the fitness reaches a maximum for an optimal mutation bias that depends on population size: small populations attain larger fitness if they evolve with AT mutation bias; intermediate populations attain maximum fitness if they evolve with GC mutation bias; and very large populations prefer the absence of bias, i.e., GC = 0.5; see Figure 6. Mutation bias is under genetic control, since it is determined by the genes involved in genome replication and DNA repair, and it is broadly distributed in bacterial families. In particular, obligatory intracellular bacteria, characterized by a small effective population size, due to the bottlenecks in the transmission from one host to another, tend to possess strong AT bias, usually due to the loss of repair genes. The results reported above suggest that changes in mutation patterns are not selectively neutral, but they can strongly influence the balance between unfolding and misfolding stability and the fitness of clones of the same bacterial population evolving with different mutation biases. If these clones come to compete for common resources in a meta-population scenario, we may expect that the optimal mutation bias will be selected. This reasoning suggests that one of the forces that shape the evolution of mutation bias in bacteria is meta-population selection of the resulting stability of the proteins that evolve under the specified bias [79]. Of course, experimental tests are needed to evaluate this hypothesis against alternative hypothesis on the evolution of GC bias, recently reviewed in [80].

## 11. Protein Functional Dynamics with the Elastic Network Model

The previous part of this review concerns protein folding stability, modeling proteins as static entities and representing the native state as an individual contact matrix. However, proteins are extremely dynamic, and even in the native state, they explore a fairly large amount of configuration space. As mentioned above, it is often assumed that this configuration entropy can be ignored, since it is expected to have similar values in all compact conformations, both native and non-native, so that its contribution to stability is negligible [6]. Nevertheless, the investigation of protein dynamics in the native state can yield interesting insights into protein function, and it is an active field of research.

The simplest model that can analytically predict the intrinsic dynamics of proteins in the native state is the elastic network model (ENM) [48], proposed by Tirion in 1996 [81]. The ENM belongs to the category of Go models, built from the experimental knowledge of the native state [82] (see [5] for a recent review). In Go models, instead of deriving the native state by minimizing the free energy of a given force field, a procedure that is computationally unfeasible, except for small peptides, and that can lead to errors, due to inaccuracy in the force fields, one builds the force field from the requirement that the known native structure sits at the minimum energy and that the molecule is minimally frustrated [83], *i.e.*, all native interactions are stabilizing and all non-native interactions can be neglected. The ENM force field is defined as:

(8) $$E(r_{1}\cdots r_{L})=\sum_{ij}C_{ij}^{\text{nat}}f(r_{ij},r_{ij}^{\text{nat}})$$

where *C_ij_* is the contact matrix, *r_ij_* is the distance between atoms *i* and *j* and *f* describes a pairwise interaction that attains its minimum when $r_{ij}=r_{ij}^{\text{nat}}$. For simplicity, it is customary to assume that *f* does not depend on the type of atoms, but only on the distance, $r_{ij}^{\text{nat}}$. The ENM can be analytically studied in a harmonic approximation that only considers small displacements from the equilibrium positions, $r_{ij}^{\text{nat}}$. To second order in the displacements, the effective energy is given by:

(9) $$E(r_{1}\cdots r_{L})\approx \frac{1}{2}\sum_{ij}C_{ij}^{\text{nat}}\kappa {\left(r_{ij}^{\text{nat}}\right)}^{-\gamma }{\left(r_{ij}-r_{ij}^{\text{nat}}\right)}^{2}$$

where we assume that $f^{\prime \prime }\left(r_{ij}^{\text{nat}}\right)\approx \kappa {\left(r_{ij}^{\text{nat}}\right)}^{-\gamma }$ represents the force constant of the native interaction, *ij*. The statistical mechanics of this model can be exactly computed through normal mode analysis. Normal modes are independent perturbations of the equilibrium configuration that constitute a complete set of vectors. They can be computed by diagonalizing the Hessian matrix of the second derivatives of the energy with respect to the coordinate systems. If we use degrees of freedom that are not Cartesian, such as internal coordinates, we have to consider the generalized eigenvalue equation $H\upsilon^{\alpha }=\omega_{\alpha }^{2}T\upsilon^{\alpha }$, where *H* is the Hessian matrix in internal coordinates, *T* is the kinetic energy matrix and *ω_α_* is the frequency of normal mode *α*. By the equipartition theorem, the average energy of the system distributes uniformly across all normal modes. Since the energy associated with a normal mode is the product of its squared frequency times its mean square displacements, the contribution of a normal mode to the intrinsic motion of the molecule decreases with its frequency, ${\langle \delta r^{2}\rangle }_{\alpha }=k_{B}T/\omega_{\alpha }^{2}$, *i.e.*, low energy normal modes generate larger internal fluctuations.

Surprisingly, despite normal modes being only valid for very small displacements, it has been observed that low frequency normal modes that describe the collective motions of proteins, such as inter-domain motion, correlate very well with observed functional conformation changes [84,85]. In particular, some functional conformation changes even up to several Å are well represented by a few low-frequency, collective normal modes. Is this correlation a result of the physical laws or natural selection? To answer this question, we need a null model of the conformation change that would be expected under a generic perturbation, for instance due to the binding of the ligand. We proposed in [86] a null model based on linear response theory that assumes that the contribution of the normal mode, *α*, to a generic conformation change, $c_{\alpha }^{2}$, is proportional to its contribution to the thermal dynamics, $c_{\alpha }^{2}\propto \omega_{\alpha }^{-2}$. We tested this null model in [87], finding that it agrees well with observations, since the correlation between $c_{\alpha }^{2}$ and $\omega_{\alpha }^{-2}$ is significant and large for almost all conformation changes present in the PDB larger than 1 Å. This result is not trivial, since small conformation changes, which are dominated by the experimental error, show much smaller correlations.

Based on this null model, we can then measure excess correlations between $c_{\alpha }^{2}$ and $\omega_{\alpha }^{-2}$ through the parameter $\rho =\text{Corr}({\left(c_{\alpha }\omega_{\alpha }\right)}^{2},\omega_{\alpha }^{-2})$. If *ρ* is significantly positive, low-frequency normal modes contribute to the conformation change more than expected based on the null model. In turn, this has the effect of reducing the harmonic energy barrier with respect to a conformation change that obeys the null model and has the same RMSD. On the contrary, if *ρ* is significantly negative, low-frequency normal modes are underrepresented with respect to the null model, and the harmonic energy barrier is larger than expected. Therefore, significant *ρ* > 0 suggests that natural selection acted on the intrinsic dynamics of the native state in such a way as to favor functional motions. We found that most conformation changes in the PDB are well described by the null model, but a large number of conformation changes has significant *ρ* > 0, in particular those involving the formation of homo-oligomeric protein complexes, proteins that transport ligands and phosphorylated proteins [87]. Conversely, significantly negative *ρ* can be interpreted as the result of natural selection to avoid a particular conformation change from happening spontaneously. Notably, we find some examples of *ρ* < 0 in regulatory proteins, for instance for conformation changes upon phosphorylation [87].

## 12. Conclusions

Simple models of protein folding and dynamics allow one to propose a hypothesis on which quantities may be the target of natural selection acting on protein stability and dynamics. These hypothesis can be tested by comparing statistical observations made on representative sets of natural proteins with suitably built null models. As a result, we can ultimately detect the fingerprint of natural selection acting on the positive and negative design of protein folding stability, protein folding rates and the intrinsic motion of proteins. Through these studies, a synergy between physics and biology is created: evolutionary arguments allow one to test and better understand protein folding, and protein folding models allow one to better understand and model evolution.
